# Exploration of the Dual Role of Dectin-1 in Tumor Development and Its Therapeutic Potential

**DOI:** 10.3390/curroncol31110536

**Published:** 2024-11-17

**Authors:** Yuxuan Cai, Ke Wu

**Affiliations:** Department of Gastrointestinal Surgery, Tongji Medical College, Union Hospital, Huazhong University of Science and Technology, Wuhan 430022, China; m202276039@hust.edu.cn

**Keywords:** Dectin-1, tumor immunology, tumor microenvironment, targeted therapy, pattern recognition receptor

## Abstract

Immunotherapy, particularly immune checkpoint inhibitors like PD-1, PD-L1, and CTLA-4, has revolutionized cancer treatment. However, the role of the innate immune system, especially pattern recognition receptors, in cancer development and immunity is gaining more and more attention. Dectin-1, a C-type lectin receptor primarily involved in antifungal immunity, has emerged as a significant player in cancer biology, exhibiting both pro-tumor and anti-tumor roles. This dual function largely depends on the tumor type and microenvironment. Dectin-1 can promote immune responses against tumors like melanoma and breast cancer by enhancing both innate and adaptive immunity. However, in tumors like pancreatic ductal adenocarcinoma and colorectal cancer, Dectin-1 activation suppresses T cell immunity, facilitating tumor progression. This review explores the complex mechanisms by which Dectin-1 modulates the tumor microenvironment and discusses its potential as a therapeutic target for cancer treatment.

## 1. Introduction

Cancer remains a significant health burden worldwide, with a rising incidence and mortality rates in the United States [1]. Despite advances in treatment options, including surgery, chemotherapy, and radiation therapy, there is a critical need for novel therapeutic strategies that effectively target tumors while minimizing adverse effects on normal tissues. Immunotherapy, which utilizes the body’s immune system to recognize and eliminate cancer cells, has emerged as a promising approach in the fight against cancer. Immune checkpoint inhibitors (PD-1, PD-L1, and CTLA-4), developed from a deep understanding of T cell biology, have achieved varying degrees of success in treating many types of cancer [2]. As monotherapies or in combination with other anti-tumor agents, these inhibitors have revolutionized the management of advanced malignancies such as melanoma [3], renal cell carcinoma [4], and non-small cell lung cancer [5]. Some tumors that were once considered inoperable now have the potential for R0 resection, thanks in part to the role of immune checkpoint inhibitors (ICIs).

A complex interaction exists between the innate immune system and tumor cells. As the body’s first line of defense against tumors, the innate immune system plays a crucial role in tumor onset, development, and anti-tumor immunity. Dysregulation of the innate immune system is associated with at least one-third of cancers and serves as a critical initiator and maintainer of oncogenic inflammation, which is closely related to tumor occurrence, development, and treatment resistance.

Innate immune cells utilize surface or intracellular receptors that recognize evolutionarily conserved structures known as PRRs to respond to microbial invasions and self-tissue damage. Substances exposed or released during cellular damage or death, or by foreign invaders, are referred to as pathogen-associated molecular patterns (PAMPs) and damage-associated molecular patterns (DAMPs) [6]. The binding of these substances to PRRs initiates complex signal transduction pathways, activating innate immune cells to produce phagocytic effects or release inflammatory mediators, thereby eliminating external invaders and restoring homeostasis. Research over the past decade has illuminated the intricate and diverse roles of PRR signaling in innate immune cells concerning the body’s anti-tumor immunity. Specific PRRs can exhibit contrasting anti-tumor or pro-tumor effects in different environments, highlighting a significant challenge in developing targeted anti-tumor drugs that act on the innate immune system.

Dectin-1, a key C-type lectin receptor, plays a vital role in the innate immune system’s defense against fungal infections. Structurally, it is a single-chain transmembrane protein, with its N-terminal located extracellularly and the C-terminal intracellularly. The extracellular region contains a carbohydrate recognition domain (CRD) that primarily binds to carbohydrate structures, such as β-glucan, a major component of fungal cell walls. The intracellular region contains a sequence known as the “Immunoreceptor Tyrosine-based Activation Motif” (ITAM), which is crucial for its signal transduction function [7]. Upon ligand recognition, Dectin-1 undergoes a conformational change that triggers the clustering of hemITAMs located at its cytoplasmic tail. This clustering event leads to the phosphorylation of the two tyrosine residues within the hemITAM motifs by Src family kinases, creating binding sites for Syk kinases and initiating subsequent signaling cascades [8]. Once activated, Syk initiates diverse signaling pathways: it activates the PI3K/AKT pathway to induce the production of reactive oxygen species (ROS) [9]; it induces an elevation in intracellular calcium levels by activating PLC-γ2, resulting in the activation of Nuclear Factor of Activated T cells (NFAT) and subsequent expression of Early Growth Response transcription factors (Egr), COX-2, and IL-2 [10]. Moreover, Syk promotes the formation of the Syk/CARD9/BCL10/MALT1 complex, activating NF-κB and leading to the expression of pro-inflammatory cytokines such as IL-1β, IL-6, and TNF-α [11]. Additionally, Syk can activate the interferon pathway through CARD9 to bolster protective immune responses, including the promotion of M1 polarization in macrophages [12,13].

Furthermore, Dectin-1’s signaling pathway synergizes with Toll-like receptors (TLRs), enhancing the production of pro-inflammatory cytokines and promoting a more effective immune response against pathogens [14].

Beyond its role in initiating inflammation, Dectin-1 connects innate immunity to the adaptive immune system. It modulates T helper cell differentiation, particularly favoring the Th17 response, which is vital for controlling fungal infections [14]. Additionally, Dectin-1 engagement on dendritic cells enhances antigen presentation and the subsequent development of fungal-specific T cell responses [15]. Recently, Dectin-1 has emerged as a significant player in cancer biology, characterized by a delicate balance between promoting effective anti-tumor immune responses and facilitating tumor growth and immune evasion. This article aims to discuss in detail the dual role of Dectin-1 in tumor development and current efforts to use it as a therapeutic target for tumor management. It serves as a platform for exploring the therapeutic potential of Dectin-1 in cancer therapy.

## 2. The Dual Role of Dectin-1 in Cancer

Recent research indicates that Dectin-1, similar to other PRRs in tumor contexts, plays a dual role, acting both as a tumor suppressor and promoter, depending on tumor type and microenvironmental heterogeneity. In melanoma [16], multiple myeloma [17], and breast cancer [18], the activation of the Dectin-1 signaling pathway correlates with effective induction of anti-tumor innate and adaptive immunity.

Conversely, in pancreatic ductal adenocarcinoma (PDAC) [19], colorectal cancer (CRC) [20], and gastric cancer (GC) [21], Dectin-1 signaling activation is associated with the suppression of adaptive immunity and tumor progression. In pancreatic ductal adenocarcinoma models, Dectin-1 activation promotes the production of tolerogenic macrophages and suppresses adaptive immunity, facilitating tumor progression [19]. Conversely, the co-activation of Dectin-1 and the TNF receptor superfamily member CD40 with agonists alters the phenotype of pro-tumoral Tumor-Associated Macrophages (TAMs) within the tumor microenvironment, enhancing anti-tumor immunity and impeding tumor progression [22]. This underscores the substantial flexibility of Dectin-1 in modulating the anti-tumoral or pro-tumoral characteristics of immune cells, influenced by factors such as membrane molecule interactions.

**Figure 1:**
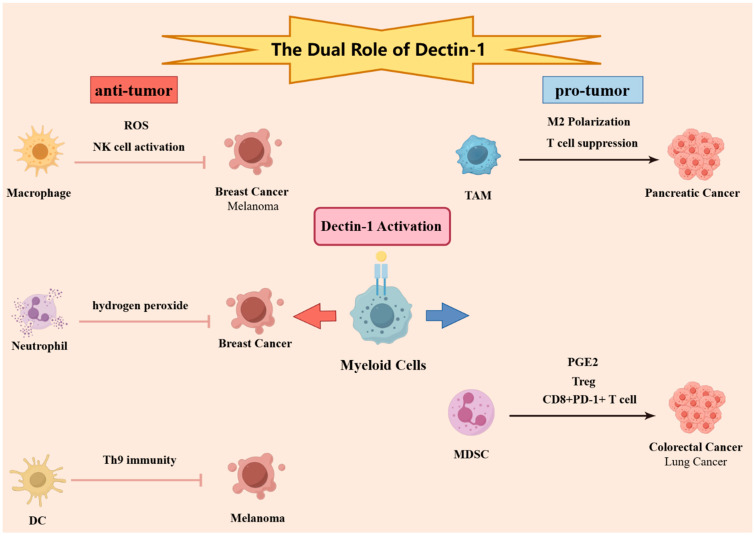
The Dual Role of Dectin-1 in Tumor Biology.

The mechanisms underlying Dectin-1’s anti-tumoral and pro-tumoral effects are complex, involving various innate and adaptive immune cell types, intercellular interactions, tumor recognition by immune cells, immune cell reprogramming, and microenvironmental regulation. These processes encompass diverse biological functions and activation of multiple signaling pathways, with significant fungal influences within the tumor microenvironment and even individual aging. Figure 1 displays a graphic abstract of the dual role of dectin-1 in tumor biology. Subsequent sections will delve into these aspects based on current research.

Dectin-1 is activated in various tumor microenvironments and different immune cells, resulting in either anti-tumor or pro-tumor effects depending on the context. In breast cancer and melanoma, the activation of Dectin-1 can lead to anti-tumor effects. In breast cancer, the activation of macrophage Dectin-1 promotes the production of reactive oxygen species (ROS), thereby inhibiting tumor growth, while the activation of neutrophil Dectin-1 enhances hydrogen peroxide production, contributing to tumor suppression. In melanoma, the activation of macrophage Dectin-1 facilitates NK cell-mediated tumor killing through the activation of NK cells. Additionally, the activation of dendritic cell (DC) Dectin-1 promotes Th9 differentiation, thereby enhancing Th9-mediated anti-tumor responses. Conversely, in pancreatic cancer, colorectal cancer, and lung cancer, the activation of Dectin-1 can lead to pro-tumor effects. In pancreatic cancer, the activation of tumor-associated macrophage (TAM) Dectin-1 promotes the M2 polarization of TAMs, inhibiting T cell immunity and thus facilitating tumor progression. In colorectal cancer, the activation of myeloid-derived suppressor cell (MDSC) Dectin-1 enhances the production of PGE2, promoting tumor growth. In lung cancer, the activation of MDSC Dectin-1 promotes tumor progression by increasing the proportion of Tregs and CD8+PD-1+ exhausted T cells within the tumor microenvironment (TME).

### 2.1. The Anti-Tumor Role of Dectin-1

The anti-tumor activity of Dectin-1 is mediated through various innate immune cells, including dendritic cells (DCs), macrophages, NK cells, and neutrophils, as well as different subtypes of adaptive immune cells. This primarily involves the direct cytotoxic effects of innate immune cells on tumors and their regulatory role on anti-tumor adaptive immunity. Dectin-1 signaling is linked to the anti-tumor activity of macrophages. The expression of mannose receptors (MRs) and Dectin-1 on macrophages is upregulated in response to IL-13. These receptors can recognize sialic acid structures on tumor surfaces, activating downstream Syk-Neutrophil cytosolic factor 1 (P47phox) signaling pathways and the arachidonic acid (AA)-12 and 15-lipoxygenase (HETE)-peroxisome proliferator-activated receptor γ (PPARγ) signaling axis. This leads to the generation of reactive oxygen species (ROS) and L-arginine-related suppressive effects in mouse T cell lymphoma, ovarian cancer, and breast cancer cells [18]. Dectin-1 enhances the tumor-killing activity of neutrophils. Neutrophils can mediate tumor cytotoxicity by recognizing Nidogen-1 on the surface of mouse breast cancer cells AT3 via Dectin-1, and this effect does not depend on the activation of the Syk signaling pathway. Specifically, in vitro studies demonstrated that antibodies targeting Clec4e and Dectin-1 significantly reduced neutrophil-mediated cytotoxicity against both AT3 and LLC tumor cells, while antibodies against NKG2D showed no effect. Furthermore, treatment with the SYK inhibitor R788 did not affect the tumor cytotoxicity of neutrophils, indicating that SYK-dependent signaling is not essential for this process. Dectin-1 also enhances the tumor-killing effect mediated by the interaction between neutrophil Cathepsin and tumor G-rage. This suggests that Dectin-1 activation not only directly activates the cytotoxic signaling pathways of neutrophils but also strengthens the neutrophil–tumor killing synapse [23]. Dectin-1 signaling mediates communication between DCs, macrophages, and NK cells, inducing NK-cell-mediated tumor cytotoxicity. DCs and macrophages can enhance the tumor-killing activity of NK cells by recognizing N-glycan on the surface of melanoma B16 cells, which relies on the Dectin-1-IRF5-lnma signaling axis. In vitro experiments showed that in the co-culture killing system of NK cells, splenocytes, and B16 tumor cells, CD11b+CD11c+ cells from Dectin-1-/- mice significantly reduced the tumor-killing activity of wild-type (WT) NK cells compared to the control group. In vivo, in the B16 metastatic model and subcutaneous model, the knockout of Dectin-1 promoted tumor growth and progression. However, after depleting NK cells, no significant difference in tumor progression was observed between the two groups. The expression of N-glycan on the surface of different tumor types is heterogeneous, and the varying binding ability of Dectin-1 to various tumor N-glycan structures may lead to differences in DC, macrophage-induced, and NK-mediated tumoricidal effects [16]. The Dectin-1 on the surface of macrophages also mediates, alongside its surface tetraspan protein MS4A4A, the NK cell-dependent control of the metastasis of highly N-glycosylated melanoma B16F1 cells. Mechanistically, MS4A4A is involved in generating appropriate phosphorylation of Syk during Dectin-1 activation and the production of downstream inflammatory cytokines and reactive oxygen species, while also participating in macrophage recognition of NK cells, thus promoting NK cell tumor cytotoxicity [24]. The Dectin-1 signaling of DCs and macrophages also mediates the connection between innate immunity and adaptive anti-tumor immunity. The activation of DC Dectin-1 promotes anti-tumor Th9 immunity. Specifically, in vitro studies have shown that bone-marrow-derived dendritic cells (BMDCs) and spleen CD11c+ cells stimulated with curdlan enhance Th9 differentiation. Compared to BMDCs matured using traditional methods, DCs matured with curdlan exhibit increased expression levels of co-stimulatory molecules, CD86/80, CD40, and particularly TNFSF15 and OX40L. The upregulation of these molecules is dependent on Dectin-1 and specifically promotes Th9 differentiation, with no significant effects on the differentiation of Th1, Th2, Th17, and Tregs. In mouse melanoma and myeloma subcutaneous tumor models, the immunization of mice with Dectin-1-activated DCs induced potential Th9 and IL-9-dependent anti-tumor immune responses. Mechanistically, curdlan activates the Syk-raf1 and NF-κB pathways, promoting the nuclear translocation of p50 and p50-RelB, and upregulating the expression of TNFSF15 and OX40L, thereby promoting Th9 differentiation [17].

The activation of Dectin-1 signaling in DCs is involved in reversing the immunosuppressive tumor microenvironment and inhibiting tumor progression. In a mouse model of breast cancer, curdlan-activated DCs were reprogrammed and acquired resistance to cancer cell-derived thymic stromal lymphopoietin (TSLP). These cells reversed the tumor-promoting environment dominated by iTh2, promoted the survival of Th1 cells, and inhibited tumor progression through the production of IL-12bp. Unlike DCs activated through TLR-7/8 or poly I:C, DCs activated through Dectin-1 can induce CD8+ T cells to express CD103 (integrin αE), a ligand for cancer cell E-cadherin. The activation of DC Dectin-1 through DC-derived integrin αVβ8 and TGF-β activates these mucosal CD8+ T cells, leading to their retention and accumulation in tumors, which increases cancer cell necrosis and inhibits established breast cancer that grows independently of the tumor microenvironment [25]. In an immune checkpoint inhibitor-resistant PDAC mouse model, systemic combined administration of the Dectin-1 stimulant β-glucan and CD40 agonistic antibody eliminated tumors and induced immune memory. Notably, this effect depends on cDC1 and T cells, but not on classic T cell cytotoxicity or the immune checkpoint blockade. Combined administration induced T cell-mediated IFN-γ signaling and promoted the anti-tumor phenotype of TAMs [22].

Additionally, Dectin-1 regulates tumor development by interfering with the signaling of other pattern recognition receptors. A study showed that Dectin-1 is upregulated in liver fibrosis and liver cancer, but its absence exacerbates liver fibrotic inflammation and accelerates oncogenesis. Mechanistically, Dectin-1 activation led to the downregulation of TLR4 and its co-receptor CD14, thereby undermining TLR4 signaling in hepatic stellate cells and inhibiting liver fibrosis and oncogenesis [26].

Collectively, these results suggest that Dectin-1 activation mediates anti-tumor effects through complex immunological and cytobiological mechanisms, including inflammation, immune microenvironment modulation, adaptive immunity regulation, interactions between membrane molecules, and intricate intracellular signal transduction.

Table 1 provides a concise summary of the anti-tumor effects of Dectin-1 in melanoma, breast cancer, and multiple myeloma, as well as its pro-tumor effects in pancreatic ductal adenocarcinoma, colorectal cancer, gastric cancer, renal clear cell carcinoma, and lung adenocarcinoma, along with the underlying mechanisms involved.

### 2.2. The Pro-Tumor Role of Dectin-1

In recent years, an increasing number of studies have focused on the pro-tumor effect of Dectin-1. A high expression of Dectin-1 in renal clear cell carcinoma [28], lung adenocarcinoma [29], colon cancer [20], and gastric cancer [21] is associated with poor prognosis in patients. As a pattern recognition receptor mainly expressed on the surface of myeloid cells, Dectin-1 is preferentially expressed in tumor-associated macrophages (TAMs) and myeloid-derived suppressor cells (MDSCs), which exist in the immunosuppressive tumor microenvironment and contribute to the inhibition of anti-tumor immunity. Specifically, Dectin-1 signaling induces the proliferation and activation of immune-suppressive TAMs and reprograms MDSCs, thereby inhibiting adaptive anti-tumor immunity and promoting tumor progression. A study demonstrated that in a mouse lung adenocarcinoma model, the intratumoral fungus Aspergillus sydowii activates MDSCs through the Dectin-1/CARD9/IL-1β pathway via β-glucan, mediating the activation of MDSCs and increasing the proportion of Tregs and PD-1+CD8+ T cells in the tumor microenvironment, thereby promoting tumor progression [29]. This study suggests that fungi can regulate the immune microenvironment through interaction with Dectin-1 on immune cells. Another study indicated that in a mouse colorectal cancer model, Dectin-1 signaling induces the production of MDSC PGE2, suppressing the production of IL-22BP and promoting tumor progression. Knocking out Dectin-1 significantly inhibits the development of colorectal tumors induced by AOM-DSS, regardless of the presence of intestinal commensal microbiota. This tumor-suppressive effect was observed in Dectin-1-/- mice, antibiotic-treated Dectin-1-/- mice, and germ-free Dectin-1-/- mice [20]. Regarding TAMs, a study indicated that Dectin-1 expression is increased on TAMs in the tumor microenvironment of PDAC, promoting tumor progression by inducing the generation of tolerant macrophages and inhibiting adaptive immunity through binding with galectin-9. Disruption of the Dectin-1-galectin-9 signaling axis restores the anti-tumor activity of CD4+ and CD8+ T cells [19]. Additionally, Dectin-1 expression is upregulated in TAMs in human gastric cancer tissues, correlating with the decreased functionality of effector T cells. Specifically, Dectin-1+ TAMs exhibit an immunosuppressive phenotype. In subsets with a high proportion of Dectin-1+ TAMs, CD4+ T cells show decreased IFN-γ expression and increased IL-4 expression, leading to a reduced Th1/Th2 ratio. Meanwhile, CD8+ T cells exhibit decreased expression of perforin and granzyme. The expression of inhibitory receptors on CD4+ and CD8+ T cells, such as PD-1, TIM-3, and LAG-3, is increased, while CTLA4 and TIGIT expression shows no significant difference. The blockade of Dectin-1 enhances the expression of pro-inflammatory factors by Dectin-1+ TAMs, downregulates immunosuppressive factors such as LAP and Arg1, reactivates T cells, and this effect is further enhanced when combined with anti-PD-1 therapy [21]. These studies suggest that the activation of TAM Dectin-1 signaling may suppress the function of anti-tumor effector T cells by promoting immunosuppressive M2-like TAM phenotypes, thereby facilitating tumor progression. Notably, a study indicated an increase in fungal burden and enhanced Dectin-1 signaling in aged mice. Knocking out Dectin-1 resulted in reduced IL-1β levels and decreased proportions of Tregs and MDSCs in oral squamous cell carcinoma in mice, leading to slower tumor progression and reduced tumor burden [30]. This indicates that Dectin-1 may be involved in the complex interplay among aging, microbiota, and tumors, providing new insights into the characteristics of the tumor microenvironment in aging individuals.

In summary, the activation of Dectin-1 in TAMs and MDSCs, possibly mediated by the Syk-CARD9 pathway and influenced by the ligand galectin-9, leads to the generation of immunosuppressive cells and the formation of an immunosuppressive tumor microenvironment, inhibiting adaptive anti-tumor immunity and promoting tumor progression.

### 2.3. Exploration of Novel Anti-Tumor Agents Targeting Dectin-1

As previously reviewed, Dectin-1, widely expressed on the surface of myeloid cells, can participate in inducing anti-tumor immunity and regulating the tumor immune microenvironment through complex mechanisms. Thus, Dectin-1 presents a promising target for cancer therapy. In recent years, researchers have focused on developing novel anti-tumor agents targeting Dectin-1 across various tumor types. The main strategies include: (1) Improving the pharmacokinetic properties of existing Dectin-1 agonists to enhance Dectin-1-dependent anti-tumor immunity; (2) interfering with Dectin-1 signaling to reverse the tumor immune-suppressive microenvironment; (3) utilizing Dectin-1 as a delivery system to target immune cells for delivering other anti-tumor agents; (4) and combining Dectin-1 with other anti-tumor agents to strengthen anti-tumor immune responses through various mechanisms. Curdlan, a β-glucan-type Dectin-1 agonist, has been shown to induce anti-tumor immunity in melanoma. However, its hydrophobicity and heterogeneity limit its consistent binding with Dectin-1. Mingming Bao et al. developed a partially oxidized curdlan derivative, β-1,3-polyglucuronic acid (PGA). The PGA-45 polymer may activate DCs by enhancing IKK-β phosphorylation and reducing phosphorylated Akt expression through binding with Dectin-1 and other cell surface molecules like TLR4, inducing co-stimulatory molecules and cytokines, and promoting the proliferation of allogeneic T cells and IL-2 expression. In vivo experiments confirmed that PGA-45 is more effective than curdlan in inducing anti-B16F10 melanoma immunity [31]. The Dectin-1/galectin-9 signaling axis plays a critical role in forming an immune-suppressive tumor microenvironment in PDAC. Wenxi Zhou et al. developed a drug delivery system composed of bone marrow mesenchymal stem cell (BM-MSC) exosomes, electroporation-loaded galectin-9 siRNA, and an oxaliplatin (OXA) prodrug-modified surface. This system disrupts the Dectin-1-galectin-9 signaling axis in PDAC by reversing the immune-suppressive phenotype of TAMs, increasing the proportion of effector T cells in the tumor, and reducing Tregs, thereby inhibiting tumor progression [27]. CpG oligodeoxynucleotides are immune activators, but their therapeutic effects are limited by degradation and insufficient cellular uptake. Huijie Zhang et al. constructed a composite NH2-Glu/CpG consisting of aminated yeast β-D-glucan (NH2-Glu) and CpG ODNs, targeting macrophages effectively. This complex significantly enhances drug uptake via Dectin-1-mediated endocytosis and synergizes the anti-tumor immune induction functions of yeast β-D-glucan and CpG ODNs, significantly inhibiting tumor growth without notable toxicity [32]. Ferumoxytol (FMT), an FDA-approved iron oxide nanoparticle, has been shown to promote M1 cell polarization, and its combination with other anti-tumor agents has a synergistic effect on inducing stronger anti-tumor immunity. Xinghan Liu et al. developed a nano-agent composed of FMT coated with β-glucan to treat mouse B16F10 melanoma. FMT acts as a drug delivery agent, stimulating anti-tumor immunity, while β-glucan, an immunomodulator targeting Dectin-1, inhibits B16F10 melanoma growth. The nano formulation upregulates Dectin-1 expression on RAW264.7 cells, induces higher levels of M1 macrophage infiltration in melanoma, enhances ROS and phagocytic activity, and is more effective than using FMT and β-glucan alone [33]. Interestingly, Dectin-1 appears to play a significant role in fungal-induced tumor resistance to radiotherapy. Stephen L. Shiao et al. found that intestinal fungi in mice with breast cancer and melanoma may weaken the effectiveness of tumor radiotherapy through interactions with host Dectin-1. Antifungal drugs can reduce tumor resistance to radiotherapy in mice, and this effect disappears in Dectin-1 knockout mice. Additionally, knocking out Dectin-1 weakens tumor resistance to radiotherapy, similar to the effects of antifungal administration. These findings suggest that Dectin-1 modulators may have a synergistic effect on radiotherapy, providing new potential therapeutic targets for tumors resistant to radiotherapy [34]. Furthermore, a Phase I trial of oral yeast-derived β-glucan to enhance anti-GD2 immunotherapy in resistant high-risk neuroblastoma found that a positive anti-mouse antibody response and Dectin-1 rs3901533 polymorphism are associated with better overall survival in patients, supporting Dectin-1 as a promising therapeutic target for human tumors [35].

In conclusion, based on the characteristics of Dectin-1-mediated immune activation and tumor microenvironment regulation, Dectin-1 represents an attractive and promising target for cancer therapy.

Table 2 briefly summarized the potential key strategies for developing tumor immunotherapy targeting Dectin-1, including specific objectives of these strategies and examples of their application.

## 3. Discussion

This review provides a comprehensive overview of Dectin-1’s dual role in tumor promotion and inhibition, alongside efforts to develop anti-tumor drugs targeting Dectin-1 based on existing research. The dual role of Dectin-1 in tumor biology may be attributed to several factors: (1) Different tumors have distinct microenvironments that can shape Dectin-1’s function; (2) various immune cells express Dectin-1, and its activation in different immune cell types can lead to diverse functional outcomes; (3) and interactions between Dectin-1 and other membrane molecules can modulate their functions, further influencing tumor progression.

Dectin-1’s function is largely shaped by the tumor microenvironment (TME), which varies significantly across cancer types. For example, in colorectal cancer, Dectin-1 promotes tumor progression by facilitating the activation of MDSCs, which are immunosuppressive and contribute to a pro-tumorigenic environment. In this context, Dectin-1 signaling enhances PGE2 production and inhibits IL-22BP generation, thereby facilitating tumor progression [20]. Conversely, in cancers where DCs are abundant in the TME, such as melanoma, Dectin-1 activation boosts cross-presentation of tumor antigens, leading to Th9 differentiation and a stronger anti-tumor response [17]. These findings indicate that the type and functional state of immune cells expressing Dectin-1 within specific TMEs play crucial roles in determining its impact on tumor development.

Dectin-1, as a C-type lectin receptor, is part of the broader family of pattern recognition receptors (PRRs), including Toll-like receptors (TLRs) and NOD-like receptors (NLRs). The functions of these molecules in tumor biology demonstrate both shared features and unique characteristics. Take TLRs as an example, the most notable differences between Dectin-1 and TLRs in tumor biology lies in (1) ligand specificity, (2) cellular expression and microenvironmental modulation, and (3) signaling pathway versatility. Firstly, DDectin-1’s ligand, β-glucan, is a relatively specific PAMP, limiting the contexts in which Dectin-1 can be activated compared to TLRs, which recognize a wide array of ligands. This specificity enables Dectin-1 to fine-tune immune responses rather than triggering broad, systemic inflammation. Secondly, Dectin-1 is predominantly expressed on the surface of myeloid cells, including macrophages, neutrophils, monocytes, and certain subsets of DCs [7]. This expression pattern allows Dectin-1 to play a central role in modulating a relatively more restricted immune response within the TME. Conversely, TLRs are expressed more widely across various immune cells, including macrophages, DCs, natural killer (NK) cells, B cells, and certain T cell subsets [36]. Each TLR subtype has a distinct expression pattern: for example, TLR4 is commonly found in macrophages and epithelial cells, whereas TLR3 is primarily expressed in DCs and fibroblasts. This widespread expression pattern enables TLRs to initiate broad immune responses that can either suppress or promote tumor development based on the specific ligands encountered and the context of their activation [36]. Thirdly, Dectin-1’s Syk-CARD9 pathway allows for complex signaling cross-talk, which can result in dual immune-modulating functions. TLRs, with their MyD88/TRIF pathways, are often more linear, leading to more defined immune outcomes [36]. Dectin-1 primarily signals through the Syk-CARD9 pathway. Upon ligand binding, Dectin-1 activates spleen tyrosine kinase (Syk), which subsequently interacts with the adaptor protein CARD9, leading to the activation of NF-κB and the production of pro-inflammatory cytokines such as TNF-α, IL-6, and IL-1β. Additionally, Dectin-1 signaling can induce the production of ROS and the maturation of DCs, enhancing the cross-presentation of tumor antigens to T cells [16]. However, in certain cancer contexts, Dectin-1 activation can lead to the recruitment of immunosuppressive cells, thereby promoting tumor progression [29]. On the contrary, TLRs signal through two main pathways: the MyD88-dependent and the TRIF-dependent pathways. Most TLRs (e.g., TLR2, TLR4, TLR5, and TLR9) utilize the MyD88 adaptor protein to activate NF-κB and MAPKs, leading to the production of pro-inflammatory cytokines. TLR3 and TLR4 can also signal via the TRIF pathway, which results in the activation of interferon regulatory factors (IRFs) and the production of type I interferons [36]. This versatility in signaling allows TLRs to induce broad immune responses, ranging from inflammation to antiviral defenses, depending on the type of TLR engaged and the co-signaling pathways activated.

Dectin-1 can also interact with various membrane molecules within the TME, which may modulate Dectin-1 signaling pathways and ultimately influence anti-tumor or tumor-promoting outcomes. MS4A4A, a tetraspan surface molecule, is expressed on macrophages and serves as one of the polarization markers for M2 macrophages and TAMs. One study demonstrated that Dectin-1 is functionally associated with MS4A4A on the lipid rafts of macrophages. MS4A4A is essential for the activation of the Syk-dependent signaling pathway upon Dectin-1 activation, as well as for the subsequent production of cytokines and ROS. Additionally, it is necessary for the Dectin-1-dependent recognition of N-glycans on tumor cells and for triggering NK cell-mediated tumor cell killing, thereby controlling tumor metastasis [24]. Another study showed that Dectin-1 also interacts with TLR4 and CD14 to alleviate liver fibrosis and oncogenesis. Mechanistically, the activation of Dectin-1 led to the downregulation of TLR4 and its co-receptor CD14, thereby, undermining the TLR4 signaling in hepatic stellate cells and inhibiting liver fibrosis and oncogenesis [26].

In conclusion, as an evolutionarily conserved pattern recognition receptor, Dectin-1 intricately regulates host–tumor interactions, either inhibiting or promoting tumor occurrence and progression. Further elucidation of the specific mechanisms governing the interaction between Dectin-1 and different tumor types is essential for the development of more effective anti-tumor therapies in the future.

## Figures and Tables

**Table 1:** The role of Dectin-1 in tumor biology and the underlying mechanisms in various tumor types.

Tumor Type	The Anti/Pro-Tumoral Role of Dectin-1	Mechanisms
Melanoma	Anti-tumor	Dectin-1 enhances the cytotoxicity of NK cells by recognizing N-glycosylated structures on the tumor surface, thereby promoting antitumor immunity. The Dectin-1 signaling pathway is associated with the IRF5-lnma axis, facilitating NK-cell-mediated tumor cell killing [16].
Breast Cancer	Anti-tumor	Dectin-1 activates macrophages through the Syk-P47phox-PPARγ signaling axis, enhancing ROS production and facilitating L-arginine-related suppressive effects on tumor cells [18].
Multiple Myeloma	Anti-tumor	Dectin-1 activates dendritic cells, enhancing their antigen-presenting capacity and promoting Th9 cell differentiation, which is dependent on the activation of the Syk-raf1 and NF-κB signaling pathways, thus augmenting adaptive immune responses [17].
Pancreatic Ductal Adenocarcinoma	Pro-tumor	Dectin-1 activation leads to the generation of tolerogenic macrophages, inhibiting adaptive immunity. The interaction between Dectin-1 and galectin-9 promotes the conversion of M2 macrophages, suppressing the activity of CD4+ and CD8+ T cells [27].
Colorectal Cancer	Pro-tumor	The Dectin-1 signaling pathway activates myeloid-derived suppressor cells, promoting the production of PGE2 and inhibiting the generation of IL-22BP, thereby facilitating tumor progression. The absence of Dectin-1 significantly suppresses tumor development [20].
Gastric Cancer	Pro-tumor	Dectin-1 is highly expressed in tumor-associated macrophages, resulting in decreased effector T cell function and promoting tumor progression. Dectin-1+ TAMs exhibit an immunosuppressive phenotype that inhibits antitumor immunity [21].
Renal Clear Cell Carcinoma	Pro-tumor	High tumoral DDectin-1 expression is an independent predictor of adverse clinical outcome in ccRCC patients, related to poorer patient RFS and OS [28].
Lung Adenocarcinoma	Pro-tumor	Dectin-1 activates MDSCs through the β-glucan/CARD9/IL-1β pathway, increasing the proportion of Tregs and PD-1+CD8+ T cells, thereby inhibiting antitumor immunity and facilitating tumor progression [29].

**Table 2:** Strategies to modulate Dectin-1 for enhanced anti-tumor immunity.

Key Strategies	Specific Objectives	Application Examples
Activation of Dectin-1	To promote anti-tumor immune responses by enhancing both innate and adaptive immunity.	Activation of Dectin-1 in breast cancer enhances the anti-tumor activity of NK cells and dendritic cells [17,18].
Combined Use of Dectin-1 Agonists	To enhance anti-tumor immune responses through a combination with other immune stimulators, such as CD40 agonists.	In models of pancreatic ductal adenocarcinoma, the Dectin-1 agonist β-glucan combined with CD40 agonists eradicated tumors and induced immune memory [22].
Interference with Dectin-1 Signaling	To reverse the tumor-immunosuppressive microenvironment and restore anti-tumor immunity.	Interfering with the signaling pathway between Dectin-1 and galectin-9 restores the anti-tumor activity of T cells, inhibiting the progression of PDAC [27].
Drug Delivery Systems	To design drug delivery systems targeting Dectin-1 to enhance the efficacy of anti-tumor drugs.	Utilizing bone-marrow-mesenchymal-stem-cell-derived exosomes to deliver galectin-9 siRNA and oxaliplatin reverses the immunosuppressive phenotype and increases the proportion of effector T cells [27].
Improvement of Existing Dectin-1 Agonist Pharmacokinetics	To enhance the pharmacokinetic properties of Dectin-1 agonists to induce stronger anti-tumor immunity.	Development of partially oxidized curdlan derivant enhances binding to Dectin-1 and promotes immune responses against B16F10 melanoma [31].
Synergistic Use with Other Anti-tumor Drugs	To enhance anti-tumor immune responses through multiple mechanisms.	The combination of FDA-approved ferumoxytol nanoparticles with β-glucan in treating mice with B16F10 melanoma enhances M1 macrophage polarization and anti-tumor immunity [33].
